# A Prolonged Case of Severe Mpox as an Opportunistic Infection in Advanced AIDS

**DOI:** 10.7759/cureus.59947

**Published:** 2024-05-09

**Authors:** Matan S Malka, Melissa Parkinson, Jason Zucker, Jacob R McLean, Marcus R Pereira, Michael T Yin, Shauna H Gunaratne

**Affiliations:** 1 Medicine, Columbia University, New York, USA; 2 Infectious Disease, Columbia University, New York, USA; 3 Infectious Disease, Columbia University Irving Medical Center, New York, USA

**Keywords:** prolonged illness, opportunistic infections, aids, hiv, monkeypox

## Abstract

The case report discusses a 29-year-old male with advanced HIV who experienced one of the longest, confirmed cases of monkeypox (mpox) infection. Despite treatment with antivirals and supportive care, including intravenous tecovirimat and vaccinia immune globulin, the patient's condition worsened over a six-and-a-half-month period. He suffered from widespread ulcerative, necrotic lesions and multiple complications, including acute kidney injury, multi-drug resistant bacterial infections, and respiratory failure. Despite repeated treatments, including brincidofovir, the patient remained PCR-positive for monkeypox virus (MPXV) with low cycle threshold (Ct) values, indicating active infection. The case underscores the severity of mpox in immunocompromised individuals, particularly those with advanced HIV, and highlights the challenges in managing such cases. The patient's persistently low CD4 count and unsuppressed HIV viral load likely contributed to the inability to clear the virus. The report emphasizes the need for further research to optimize treatment strategies for MPXV infection, especially in people living with HIV.

## Introduction

As of January 2024, 93,030 cases of monkeypox (mpox) have been reported worldwide since the outbreak began in May 2022 [1]. Mpox is caused by the monkeypox virus (MPXV) of the orthopoxvirus (OPXV) genus. Persons with HIV (PWH) have been disproportionately affected by the virus, and those with low CD4 counts have accounted for some of the most severe, necrotizing forms of the disease [2-4]. To our knowledge, this case report describes one of the longest, confirmed mpox infections in the setting of advanced HIV.

## Case presentation

**Figure 1:**
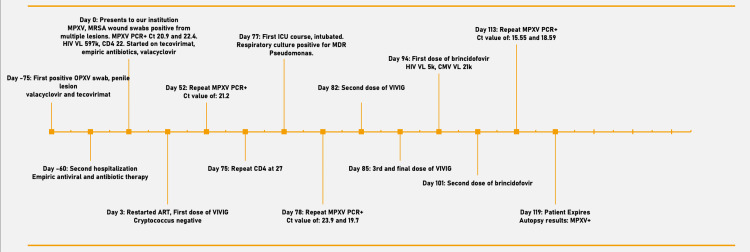
Patient timeline OPXV: Orthopoxvirus; MPXV: Monkeypox virus; MRSA: Methicillin-resistant Staphylococcus aureus; PCR: Polymerase chain reaction; Ct: Cycle threshold; ART: Anti-retroviral therapy; VIVIG: Vaccinia intravenous immune globulin; ICU: Intensive care unit; MDR: Multi-drug resistant

The patient was a 29-year-old male with AIDS not on antiretroviral therapy (ART) and had a history of genital herpes. He presented to an outside hospital with painful penile lesions concerning for mpox (Figure 1). His penile lesions were OPXV positive, and he was prescribed oral tecovirimat but only completed half the treatment course. He was admitted two weeks later and treated for superimposed bacterial and viral infections for several days before leaving against medical advice.

Two months later, the patient presented to our institution with persistent, diffuse, painful, pruritic lesions and odynophagia. He had been off ART for several months and denied any contact with MPXV-infected persons or recent travel. He endorsed being sexually active only with his wife and no sexual activity at least two months prior to admission.

**Figure 2:**
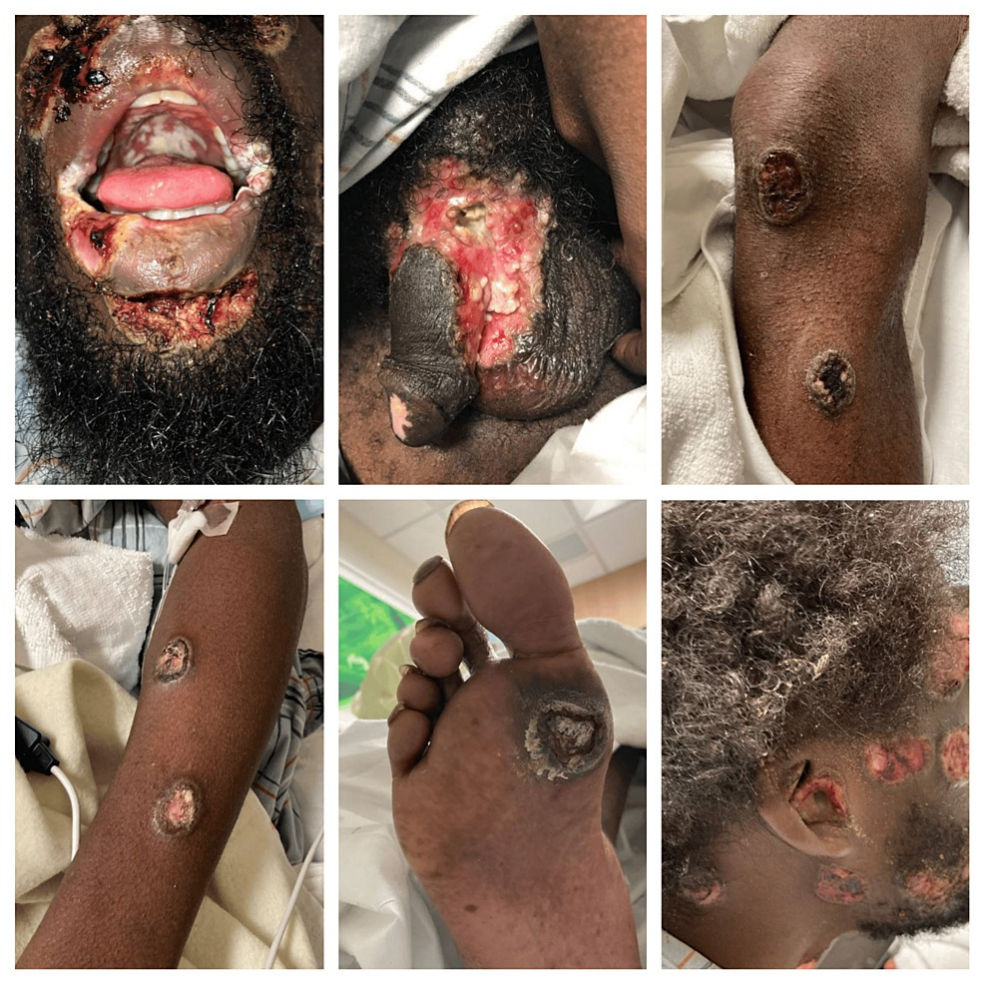
Clinical images of patient's lesions

On exam, he had widespread ulcerative, necrotic lesions on his face, scalp, trunk, and limbs (1-2 cm in diameter), with white exudate in his oropharynx (Figure 2). Additionally, there was a large, deep, purulent wound in the groin that extended around the base of the penis. The patient was admitted and started on intravenous (IV) tecovirimat due to the extent and severity of his lesions as well as broad-spectrum antimicrobial therapy.

After clinical consultation with the Centers for Disease Control and Prevention (CDC), he was treated with vaccinia immune globulin intravenous (VIGIV) on hospital day three. A tissue biopsy of his right foot and left arm lesions showed positive immunostaining for MPXV and negative stains for HSV/VZV and human herpes virus-8 (HHV-8). Perineal and scrotal wound cultures were positive for methicillin-resistant Staphylococcus aureus (MRSA) and Streptococcus dysgalactiae. The penile lesion was HSV-2 positive. PCR testing was performed using the RealStar Zoonotic Orthopoxvirus assay (Altona Diagnostics, Hamburg, Germany). Initial MPXV PCR swabs were positive from unspecified lesions with cycle thresholds (Ct) of 20.9 and 22.4 copies. The patient was also started on topical cidofovir for nasal and ear lesions. Trifluridine eye drops were started empirically and discontinued after ophthalmology did not find evidence of eye involvement. At the start of this admission, his HIV viral load (VL) was 597,000 copies/mL, and his CD4 count was 22 cells/uL. His home ART, bictegravir, emtricitabine, and tenofovir alafenamide, was reinitiated on hospital day three.

His condition worsened with acute kidney injury requiring hemodialysis. Kidney biopsy revealed severe tubular injury and mild to moderate glomerulopathy associated with HIV. No MPXV stains were performed on renal tissue. He also had CMV viremia treated with ganciclovir and polymicrobial bloodstream infections (MRSA, Serratia marcescens, Enterococcus faecium) treated with appropriate antibiotics. Throughout his hospital course, his HIV viral load remained unsuppressed with a nadir of 405 copies/mL on hospital day 113. His CD4 count was also consistently low with a peak of 27 on hospital day 75. There were concerns from the treatment team regarding the patient’s adherence to oral medications, including his ART, during his hospital stay. HIV RNA genotyping sequencing showed reverse transcriptase (RT) resistance-associated mutations (RAMs) K70Q and M184V, and integrase strand transfer inhibitor (INSTI) RAM R263K/R conferring intermediate resistance to dolutegravir. His ART was changed several times throughout admission due to these concerns and resistance.

On hospital day 77, he acutely decompensated necessitating transfer to the intensive care unit (ICU) and broad-spectrum antibiotics, vasopressors, and intubation. Tissue culture from a skin lesion on the chest grew vancomycin-resistant Enterococcus and Pseudomonas aeruginosa. Repeat lesion swabs for MPXV were positive with Cts of 23.9 and 19.7 on day 78. On hospital day 78, the respiratory culture grew multidrug resistant (MDR) Pseudomonas aeruginosa. Given his persistent severe illness and continued positive lesions for MPXV, he was given additional doses of VIGIV on hospital day 82 and day 85 and two doses of brincidofovir on hospital day 94 and day 101 after approval from the U.S. Food and Drug Administration (FDA) in conjunction with CDC consultation.

Repeat swabs were again positive for MPXV with Ct values of 15.6 and 18.6 on day 113. The patient expired on hospital day 119 from a cardiac arrest after his course was complicated by MRSA bacteremia and Candidemia, and he was made DNR given his poor prognosis. Post-mortem autopsy was notable for lung tissue positive for MPXV, Candida, and CMV on biopsy and having infarcted areas bilaterally and numerous cells with viral inclusions. Skin tissue was also positive for MPXV.

## Discussion

Severe mpox cases have been associated with prolonged illness and high rates of complications [2,4-6]. This case is particularly interesting given the patient’s prolonged course of six and a half months with continued PCR-positive skin lesions with low Cts and progressive disease. Previous studies have demonstrated prolonged MPXV positivity in patients up to 76 and 89 days, respectively [4,7]. Another case report described a prolonged course of mpox with positive saliva for MPXV at day 330, though Ct values of PCR samples decreased over time with treatment and immune reconstitution, and the remaining positive sample at day 330 had a Ct value of 35 copies [8]. Ct values of ≥ 35 copies have been shown to be associated with little to no infectivity [9], but it is still unclear whether this applies to all Ct platforms. Our patient is unique in that he had positive skin lesions with low Cts throughout his course indicating active infection on repeat testing on day 199 and day 205 after symptom onset.

Disseminated, necrotizing mpox infections like that experienced by our patient have been described in several case series among PWH with low CD4 counts and high viral loads [2,4]. These cases highlight the severity of mpox in immunocompromised patients, and mpox as an opportunistic infection in HIV [10]. Other OPXV infections have been shown to present in a more severe and prolonged manner in immunocompromised patients [11,12]. A 2022 report by the CDC on severe mpox in hospitalized patients found that among 57 patients with severe mpox, 82% had HIV but only 9% were on ART prior to mpox diagnosis, with a 21% mortality rate, further highlighting the severity among PWH [2].

Treatment among these individuals, as with our case, required extended courses of antivirals against MPXV as well as re-initiation of ART [2,4,8]. Recently, the CDC released targeted clinical considerations for severe mpox, recommending the use of multiple agents upfront in severely immunocompromised patients [13]. Unfortunately, our patient did not recover during his prolonged hospitalization and suffered numerous complications secondary to his immunocompromised state. The Ct values of his positive MPXV swabs remained low despite prolonged treatment with tecoviromat, repeated doses of VIGIV, and brincidofovir, suggesting an inability to clear MPXV or possible resistance to tecovirimat, a now recognized phenomenon [14]. Our patient’s Ct values were consistently in the twenties or lower throughout his admission likely due to his persistently low CD4 and unsuppressed HIV VL. More research needs to be done to evaluate optimal treatment and duration for MPXV infection, particularly in PWH.

## Conclusions

This case highlights the complexity of managing severe mpox infection in HIV-positive individuals. Despite aggressive treatment efforts, including antiviral therapy and adjunctive measures, the patient's condition worsened, ultimately leading to a fatal outcome. The persistence of active mpox infection, coupled with the patient's underlying immunocompromised state, underscores the need for optimized therapeutic strategies and emphasizes the importance of early and effective antiretroviral therapy in HIV/AIDS management. Further research is needed to better understand and address the challenges posed by severe mpox infection in immunocompromised patients.
